# Interrupted Time-Series Analysis of Stereotactic Radiosurgery for Brain Metastases Before and After the Affordable Care Act

**DOI:** 10.7759/cureus.21338

**Published:** 2022-01-17

**Authors:** Hind A Beydoun, Shuyan Huang, May A Beydoun, Shaker M Eid, Alan B Zonderman

**Affiliations:** 1 Research Programs, Fort Belvoir Community Hospital, Fort Belvoir, USA; 2 Intramural Research Program, National Institute on Aging, Baltimore, USA; 3 Medicine, Johns Hopkins University School of Medicine, Baltimore, USA

**Keywords:** stereotactic, radiation, metastasis, hospitalization, brain

## Abstract

The 2010 Patient Protection and Affordable Care Act was aimed at reducing healthcare costs, improving healthcare quality, and expanding health insurance coverage among uninsured individuals in the United States. We examined trends in the utilization of radiation therapies and stereotactic radiosurgery before and after its implementation among U.S. adults hospitalized with brain metastasis. Interrupted time-series analyses of data on 383,934 Nationwide Inpatient Sample hospitalizations (2005-2010 and 2011-2013) were performed, whereby yearly and quarterly cross-sectional data were evaluated and Affordable Care Act implementation was considered the main exposure variable, stratifying by patient and hospital characteristics. Overall, we observed a declining trend in radiation therapy over time, with an upward shift post-Affordable Care Act. A downward shift in radiation therapy post-Affordable Care Act was observed among Northeastern and rural hospitals, whereas an upward shift was noted among specific patient (females, 18-39 or ≥ 65 years of age, Charlson Comorbidity Index (CCI) ≥10, non-elective admissions, Medicare, self-pay, no pay or other insurance) and hospital (Midwestern, Western, non-teaching urban) subgroups. Stereotactic radiosurgery utilization among recipients of radiation therapy increased over time among Hispanics, elective admissions, and rural hospitals, whereas post-Affordable Care Act was associated with increased stereotactic radiosurgery among African-Americans and non-elective admissions and decreased stereotactic radiosurgery among elective admissions, and rural hospitals. Whereas hospitalized adults in the United States utilized less radiation therapy over the nine-year period, utilization of radiation therapy, in general, and stereotactic radiosurgery, in particular, were not consistent among distinct subgroups defined by patient and hospital characteristics, with some traditionally underserved populations more likely to receive healthcare services post-Affordable Care Act. The Affordable Care Act may be helpful at closing the gap in access to technological advances such as stereotactic radiosurgery for treating brain metastases.

## Introduction

With nearly 200,000 incident cases per year, brain metastases are the most frequently diagnosed intracranial cancers among adults in the United States [1]. The most frequent type of primary tumor resulting in brain metastases is lung cancer followed by breast, melanoma, renal, and colorectal cancer [1]. The selection of a conventional therapy for brain metastases depends on multiple health-related criteria with the overarching goal of achieving local tumor control, improving quality of life, and preventing death from neurological disease [1]. Whereas surgical resection is preferred for patients with single, operable, and/or large brain metastases causing edema or hydrocephalus, whole-brain radiotherapy (WBRT) is useful as adjuvant therapy post-surgical resection or for multiple brain metastases that cannot be surgically removed [1,2]. WBRT alone is indicated among patients with poor performance based on the Karnofsky scale or more than three brain metastases [2]. Available since the 1980s, stereotactic radiosurgery (SRS) is a minimally invasive procedure that is considered an alternative to WBRT that can quickly deliver ablative doses of radiation while minimizing radiation exposure to normal brain tissue and risks of cognitive side-effects and/or neurologic compromise [3-5]. SRS may offer a one-day treatment course with prolonged treatment planning while avoiding or delaying side effects; by contrast, WBRT is a less expensive option that reduces intracranial relapse and allows faster initiation of radiation treatment [3]. Patients having 1-3 brain metastases or good performance often receive a combination of WBRT and SRS [2], whereas patients who refuse WBRT or need salvage treatment after WBRT often receive SRS alone [2].

SRS is rapidly becoming commonplace in developed countries including the United States, Canada, and parts of Europe [6]. Recent studies have provided evidence for the cost-effectiveness of SRS among patients with brain metastases [7,8], while others have demonstrated the increasing popularity of SRS among cancer patients and especially those with brain metastases [5,9]. For instance, Guadagnolo et al. analyzed claims data from Surveillance, Epidemiology, and End Results (SEER)-Medicare and Texas Cancer Registry-Medicare (2000-2009) databases on utilization of radiation therapy among 13,488 patients diagnosed with lung, breast, prostate, colorectal, melanoma, and pancreatic cancers within their last 30 days of life [10]. Results suggested a shift towards technologically advanced treatments, with a decrease in utilization of two-dimensional radiation therapy (74.9% (2000) to 32.7% (2009)), and an increase in utilization of three-dimensional radiation therapy (27.2% to 58.5%), intensity-modulated radiation therapy (0% to 6.2%), and SRS (0% to 5.0%) [10].

The international literature suggests that expanded health insurance coverage may be among the key players in reducing healthcare disparities, especially among cancer patients [11-16]. In the United States, recent trends in SRS and non-SRS radiation therapies for brain metastases were likely influenced by the 2010 Patient Protections and Affordable Care Act (ACA) which was signed into law to reduce healthcare costs, improve healthcare quality, and expand health insurance coverage among uninsured individuals [17-20]. The ACA had multiple provisions facilitating access to cancer-related procedures among previously underserved populations, including universal coverage of eligible populations through Medicare/Medicaid, regulations that require employers with > 50 employees to provide coverage for their employees and prohibit denial of coverage because of pre-existing conditions, the Health Insurance Marketplace for individuals without employer-sponsored coverage as of October 2013, an optional Medicaid expansion for individuals 18 to 65 years of age with incomes up to 138% of the federal poverty level as of January 2014, coverage of young adults up to 26 years of age under their parents’ health insurance plan as well as coverage of all U.S. Preventive Services Task Force (USPSTF)-recommended services with A or B ratings by most private health plans or Medicare with no cost-sharing in the form of a deductible, co-payment or other out-of-pocket expense [17-19].

Recently conducted quasi-experimental studies have suggested that distinct ACA components may be responsible for increasing rates of insurance coverage among cancer patients as well as early-stage cancer diagnoses, while reducing disparities in access to needed healthcare services [21,22]. To date, studies focused on ACA in relation to the utilization of procedures aimed at prevention, diagnosis, and treatment of cancers have been scarce and have yielded inconsistent findings [17-19]. Moreover, studies examining trends in SRS utilization for treating brain metastases among U.S. adults have mainly originated from SEER-Medicare and National Cancer Database (NCD), and none specifically examined the potential role of ACA using an all-payer database at the national level. The main objective of this retrospective study is to perform interrupted time-series (ITS) analyses to examine variations in utilization of radiation therapies, in general, and SRS, in particular, among hospitalized U.S. adults diagnosed with brain metastasis using a large, all-payer, nationwide database. We hypothesized that levels and trends in utilization differed pre-post implementation of ACA components by January 2014, that patient and hospital characteristics modified the difference and/or change in utilization pre-post implementation of these ACA components.

A preprint of this article was previously posted to the Research Square server on May 13, 2020 (https://www.researchsquare.com/article/rs-27989/v1).

## Materials and methods

Data source

We performed secondary analyses of existing data from the Agency for Healthcare Research and Quality (AHRQ), Healthcare Cost and Utilization Project (HCUP), Nationwide Inpatient Sample (NIS). The AHRQ HCUP NIS is the largest publicly available, all-payer inpatient care database of community hospitals in the United States. It consists of 5-8 million hospital discharge records sampled annually from 1000 hospitals since 1988. Each year, a 20% stratified probability sample of hospitals (before 2012) or hospital discharge records (since 2012) is selected from all participating HCUP states. NIS data elements included patient demographics, up to 15 diagnoses and 15 procedures, as well as hospital course and outcomes. This study was conducted in accordance with the Declaration of Helsinki and was determined to be research not involving human subjects by Fort Belvoir Community Hospital.

Study population

The study population consists of hospital discharge records from 2005-2013 NIS databases that met inclusion criteria: 1) Age ≥ 18 years; 2) Primary or secondary diagnosis of brain metastases based on ICD-9-CM codes (191 (malignant neoplasm brain), 191.7 (malignant neo brain stem), 191.8 (malignant neo brain nec), 191.9 (malignant neo brain nos), 198.3 (sec mal neo brain/spine)). Hospital discharge records were excluded if they corresponded to patients with missing data on patient and hospital characteristics.

Study variables

Eligible discharge records were identified as corresponding to patients receiving radiation therapy if at least one of up to 15 ICD-9-CM procedure codes were the following: SRS (92.3 (stereotact radiosurgery*), 92.30 (stereo radiosurgery nos), 92.39 (stereo radiosurgery nec)) or non-SRS (92.2 (therap radiol & nucl med*), 92.21 (superficial radiation), 92.22 (orthovoltage radiation), 92.23 (radioisot teleradiother), 92.24 (teleradio using photons), 92.25 (electron teleradiotherap), 92.26 (particul teleradioth nec), 92.27 (radioactive elem implant), 92.29 (radiotherapeut proc nec), 92.31 (sing source radiosurgery), 92.32 (multisource radiosurgery), 92.33 (particulate radiosurgery)). Records that did not satisfy these criteria were identified as corresponding to patients who did not receive SRS and/or non-SRS therapies during their hospitalization. Prevalence of radiation therapy among hospitalized patients was defined as proportion of eligible records that corresponded to patients who underwent SRS and/or non-SRS treatments and prevalence of SRS was defined as proportion of eligible radiation therapy records that corresponded to patients who received SRS. Prevalence rates were examined over a nine-year study period (2005-2013) as well as before (2005-2010) and after (2011-2013) ACA implementation. Twenty-four pre-intervention (before quarter 1 of 2011) and 12 post-intervention (since quarter 1 of 2011) time points were generated using year and quarter of hospital discharge, and time trends were examined before and after stratifying by patient and hospital characteristics. Patient characteristics were sex, age, race/ethnicity, Charlson comorbidity index (CCI), admission type, and primary health insurance coverage. Hospital-level characteristics were hospital region, location/teaching status, and bed size.

Statistical analysis

All statistical analyses were conducted using STATA version 15 (StataCorp, College Station, TX), taking into account complex sampling design. First, logistic regression models were constructed for comparing distributions of patient and hospital characteristics among hospitalizations that occurred pre-post ACA implementation. Second, prevalence rates of radiation therapies and SRS were evaluated at each point in time, and trend analyses were performed by year and quarter using logistic regression. We used time point-specific prevalence rates to perform comparative ITS analyses by examining differences in levels and slopes over time between periods pre-post ACA implementation, before and after stratifying by patient or hospital characteristics [23]. ITS analysis is a robust quasi-experimental method for evaluating the impact of population-level interventions through estimation of change in outcome around the time of an intervention while avoiding individual-level unmeasured confounding and controlling for pre-existing time trends prior to that intervention [23,24]. All ITS models included indicator variables for time point (year and quarter coded as 51,…,134), post-intervention status and an interaction term between time point and post-intervention status [25]. We applied post-estimation to calculate the change in intercept and slope for pre-post ACA prevalence rates [26,27], and calculated Newey-West standard errors to adjust for autocorrelation [23,24]. We also constructed Poisson regression models whereby each patient and hospital characteristic, post-ACA status, and their interactions were examined as predictors of prevalent radiation therapy and SRS. Statistical tests were two-sided and p<0.05 was considered statistically significant.

## Results

**Figure 1:**
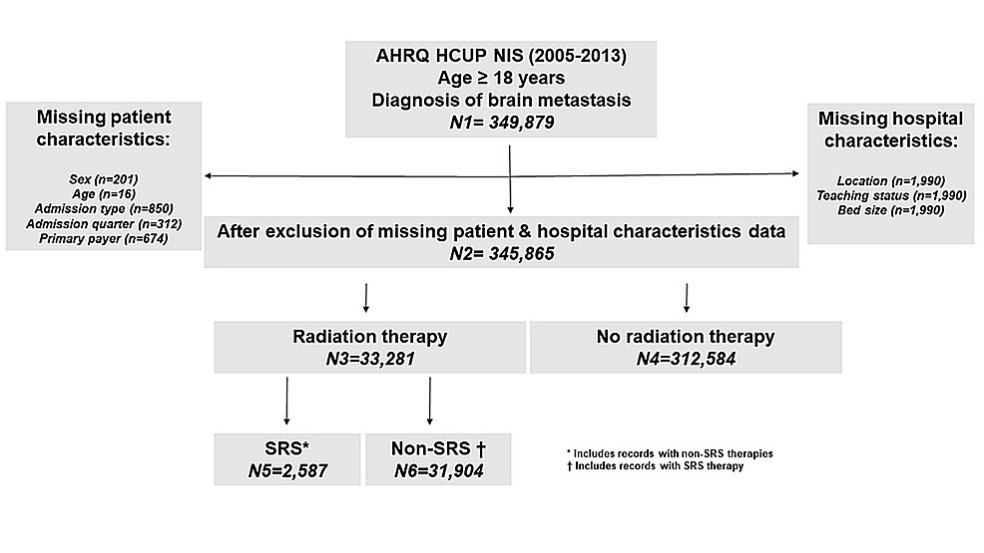
Study flowchart – Nationwide Inpatient Sample (2005-2013) AHRQ: Agency for Healthcare Research and Quality; HCUP: Healthcare Cost and Utilization Project; NIS: Nationwide Inpatient Sample; SRS: Stereotactic radiosurgery.

Of 349,879 hospitalizations identified among U.S. adults with primary or secondary diagnosis of brain metastases from 2005-2013 NIS databases, 345,865 (98.8%) were study-eligible because they had no missing data on baseline characteristics. The mean (± standard error) age of hospitalized patients with brain metastases was 61.0 (± 0.02) years and nearly 58% were <65 years of age. Of those, 228,555 (66.1%) were pre-ACA and 117,310 (33.9%) were post-ACA records. Similarly, 33,281 (9.6%) underwent any type of radiation therapy with 2,587 (7.8%) undergoing SRS and 31,904 (95.6%) undergoing non-SRS therapies, and an overlap between SRS and non-SRS therapies among 1,210 records (Figure 1).

Table 1 compares patient and hospital characteristics of eligible records pre-post ACA implementation. Whereas pre-ACA and post-ACA records did not differ by patient sex or by hospital bed size, we observed small but statistically significant differences for all remaining patient and hospital characteristics. Compared to pre-ACA records, post-ACA records were more likely to correspond to older, minority patients with CCI ≥ 10 admitted to Midwestern, Southern, or Western urban-teaching hospitals, and less likely to correspond to non-Medicare electively admitted patients.

**Table 1:** Characteristics of Brain Metastasis Patients Before and After the Affordable Care Act ACA: Affordable Care Act; aOR: Adjusted odds ratio; CI: Confidence Intervals; cOR: Crude Odds Ratio. * Adjusted odds ratios were calculated based on a fully-adjusted model for patient and hospital characteristics as predictors of pre-post Affordable Care Act status.

	Weighted %			cOR (95% CI)	aOR (95% CI)*
	Total (n=345,865)	Pre-ACA 2005-2010 (n=228,555)	Post-ACA 2011-2014 (n=117,310)		
Sex:		P=0.024			
Male	48.9	49.0	48.7	Ref.	Ref.
Female	51.1	50.9	51.4	1.02 (1.00, 1.03)	1.01 (0.99, 1.03)
Age (years):		P<0.0001			
18-39	6.9	7.1	6.7	Ref.	Ref.
40-64	50.8	51.1	50.1	1.05 (1.02, 1.08)	1.07 (1.04, 1.11)
65+	42.2	41.8	43.2	1.11 (1.08, 1.14)	1.04 (1.00, 1.08)
Race/Ethnicity:		P<0.0001			
White	65.6	61.5	70.9	Ref.	Ref.
African American	9.4	8.5	11.3	1.16 (1.13, 1.18)	1.07 (1.04, 1.09)
Hispanic	5.6	5.2	6.3	1.06 (1.03, 1.09)	1.07 (1.03, 1.09)
Other	4.6	4.2	5.3	1.09 (1.05, 1.12)	1.09 (1.05, 1.13)
Unknown	15.8	20.6	6.5	0.27 (0.26, 0.28)	0.22 (0.22, 0.23)
Charlson comorbidity index:		P<0.0001			
≤2	14.5	14.8	14.0	Ref.	Ref.
3-9	83.2	83.3	83.1	1.05 (1.03, 1.07)	1.00 (0.98, 1.03)
≥10	2.2	1.9	2.9	1.57 (1.49, 1.65)	1.38 (1.32, 1.46)
Admission type:		P<0.0001			
Non-Elective	79.6	78.9	81.0	Ref.	Ref.
Elective	20.3	21.1	18.9	0.88 (0.86, 0.89)	0.89 (0.88, 0.91)
Primary health insurance:		P<0.0001			
Medicare	43.8	42.9	45.8	Ref.	Ref.
Medicaid	11.6	11.3	12.3	1.02 (0.99, 1.04)	0.95 (0.92, 0.97)
Private insurance	38.2	39.6	35.6	0.84 (0.83, 0.86)	0.83 (0.82, 0.85)
Self-pay/No pay/Other	6.2	6.2	6.4	0.97 (0.94, 1.00)	0.92 (0.89, 0.95)
Hospital region:		P<0.0001			
Northeast	22.0	22.6	21.0	Ref.	Ref.
Midwest	23.3	23.4	23.2	1.06 (1.05, 1.08)	1.74 (1.71, 1.77)
South	36.7	36.1	37.9	1.12 (1.11, 1.14)	1.32 (1.31, 1.34)
West	17.8	17.9	17.8	1.07 (1.05, 1.08)	1.26 (1.23, 1.27)
Hospital location/teaching status:		P<0.0001			
Rural	8.5	9.1	7.5	Ref.	Ref.
Urban –Non-Teaching	33.7	35.2	30.8	1.05 (1.03, 1.07)	0.98 (0.96, 1.00)
Urban –Teaching	57.7	55.7	61.6	1.33 (1.31, 1.36)	1.33 (1.30, 1.36)
Hospital bed size:		P<0.0001			
Small	9.8	9.8	9.7	Ref.	Ref.
Medium	20.5	20.3	21.1	1.05 (1.03, 1.07)	1.01 (0.99, 1.03)
Large	69.7	69.9	69.2	1.00 (0.98, 1.01)	1.00 (0.98, 1.02)

Table 2 presents trends in utilization of radiation therapy and SRS. In general, 9.60% of brain metastasis patients underwent radiation therapies and 7.71% of brain metastasis patients who underwent radiation therapies underwent SRS. There were statistically significant trends by year and/or quarter in radiation therapy and SRS prevalence rates. Whereas the trend in radiation therapy declined over time, that of SRS is less clear-cut.

**Table 2:** Time Trends in Utilization of Radiation Therapies and Stereotactic Radiosurgery for Brain Metastasis ^a^ Of all hospitalizations corresponding to patients who were diagnosed with brain metastases; ^b^ Of all hospitalizations corresponding to patients who were diagnosed with brain metastases and underwent radiation therapies.

	% Radiation Therapy ^a^				% Stereotactic Radiosurgery ^b^			
	Quarter 1	Quarter 2	Quarter 3	Quarter 4	Quarter 1	Quarter 2	Quarter 3	Quarter 4
2005	11.71	11.94	11.10	11.0	7.41	6.65	4.95	7.08
2006	12.16	11.51	11.43	10.90	9.44	9.28	7.88	6.21
2007	11.12	11.18	10.49	10.61	7.87	7.51	7.67	7.32
2008	10.42	9.83	9.62	9.76	9.04	8.59	6.61	7.20
2009	10.32	9.25	9.39	8.80	7.52	6.32	6.12	7.71
2010	9.05	8.80	8.89	8.51	9.47	8.60	9.35	5.83
2011	8.80	9.09	8.59	8.58	9.99	8.17	7.36	7.75
2012	8.58	8.38	8.09	8.04	8.15	6.61	7.53	7.51
2013	8.14	7.61	7.67	7.52	7.51	7.64	8.80	9.48
Total	9.60				7.71			
P _trend_ (Year)	<0.0001				0.15			
P _trend_ (quarters)	<0.0001				0.002			
P _trend_ (Year & quarters)	<0.0001				0.059			

**Figure 2:**
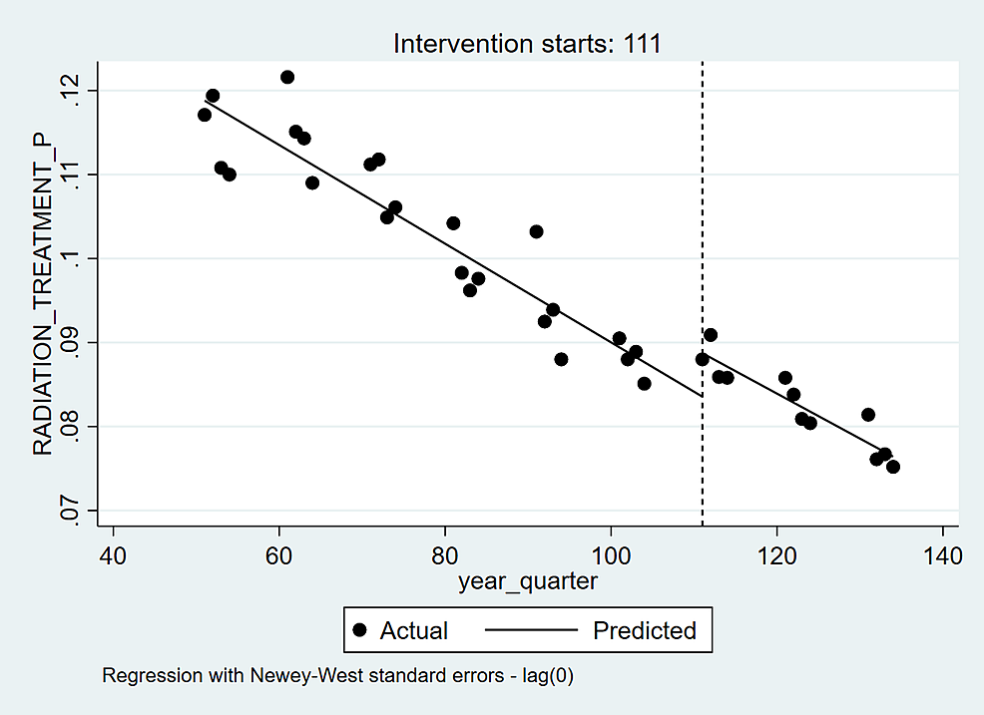
Interrupted Time-Series Analyses for Prevalence of Radiation Therapies – Nationwide Inpatient Sample (2005-2013) Note: 111 represents the 1st quarter of the year 2011.

**Figure 3:**
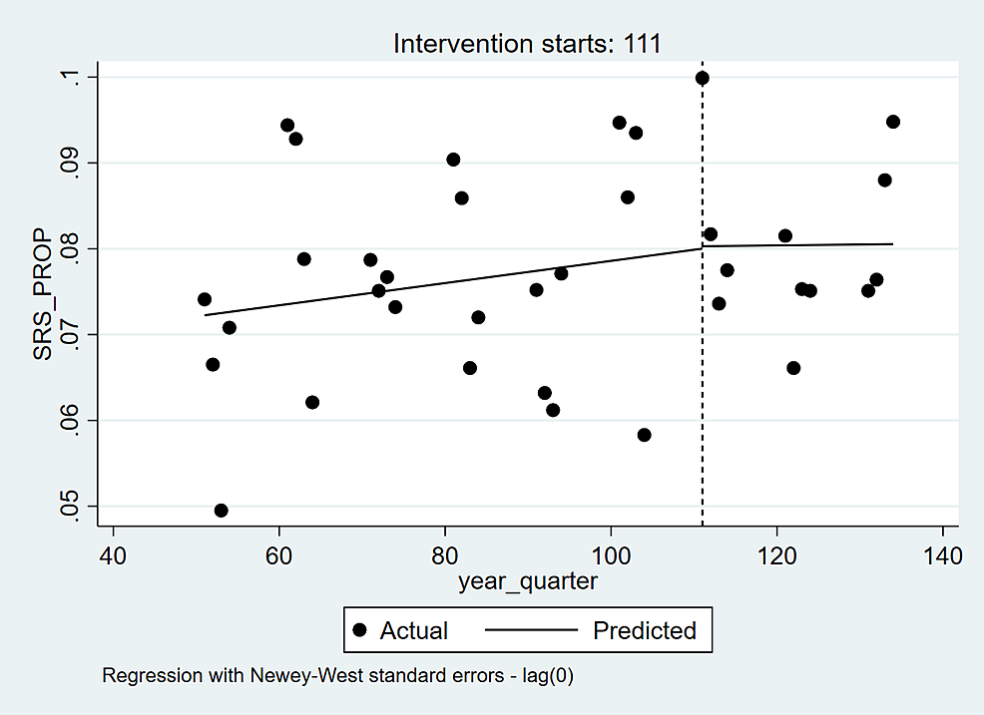
Interrupted Time-Series Analyses for Prevalence of Stereotactic Radiosurgery – Nationwide Inpatient Sample (2005-2013) Note: 111 represents the 1st quarter of the year 2011.

Table 3 and Table 4 present ITS and Poisson regression analyses for ACA as a predictor of prevalent radiation therapy and SRS. Overall, the prevalence of radiation therapy declined over time, with a significant increase in prevalence (change in intercept) around the time of ACA implementation, and no significant change in slope post-ACA (Figure 2). There was no significant change in intercept or slope with respect to SRS prevalence over time, suggesting that ACA was not related to SRS among all radiation therapy recipients (Figure 3). Poisson regression models suggested a negative relationship between post-ACA status and radiation therapy (Incidence Rate Ratio (IRR)=0.80, 95% Confidence Interval (CI): 0.78, 0.82) and a non-significant relationship between post-ACA status and SRS (IRR=1.06, 95% CI: 0.97, 1.14).

**Table 3:** Interrupted Time Series and Poisson Regression for Radiation Therapy and Stereotactic Radiosurgery Utilization – Overall and according to patient characteristics* ^a^ Of all hospitalizations corresponding to patients who were diagnosed with brain metastases; ^b^ Of all hospitalizations corresponding to patients who were diagnosed with brain metastases and underwent radiation therapies. IRR: Incidence Rate Ratio; ITSA: Interrupted Time Series Analysis; PR: Poisson Regression. * Interrupted time-series and Poisson regression analyses are presented for the total population and within strata of patient and hospital characteristics.

	Radiation Therapy ^a^				Stereotactic Radiosurgery ^b^						
	ITSA β (95% CI)			PR	ITSA β (95% CI)					PR	
	Time point effect	Post-ACA effect	Post-ACA by time effect	IRR (95% CI) P _interact_	Time point effect		Post-ACA effect	Post-ACA by time effect		IRR (95% CI) P _interact_	
Total	-.00058 (-.00069, -.00048)	.0052 (.0014, .0089)	.000052 (-.00011, .00022)	0.80 (0.78, 0.82)	.00012 (-.00022, .00048)		.00028 (-.017, .018)	-.00012 (-.00099, .00076)		1.06 (0.97, 1.14)	
Sex:											
Male	-.00056 (-.00068, -.00045)	.0038 (-.0011,.0088)	.00012 (-.00014, .00037)	0.79 (0.77, 0.82) Ref.	.00015 (-.00030, .00061)		.0021 (-.019, .024)	-.00039 (-.0012, .00038)		1.06 (0.94, 1.18) Ref.	
Female	-.00060 (-.00072, -.00048)	.0063 (.00072, .012)	0.000 (-.00031, .00029)	0.81 (0.78, 0.83) 0.664	.000099 (-.00033, .00054)		-.0011 (-.023, .021)	.00014 (-.0011, .0014)		1.06 (0.95, 1.19) 0.933	
Age (years):											
18-39	-.00044 (-.00072, -.00017)	.015 (.0029, .027)	-.00052 (-.0011, .00011)	0.84 (0.75, 0.93) Ref.	.00051 (-.00066, .0017)		-.00037 (-.065, .064)	-.0021 (-.0049, .00074)		1.01 (0.76, 1.34) Ref.	
40-64	-.00046 (-.00056, -.00036)	.0014 (-.0033, .0062)	-.000047 (-.00024, .00014)	0.81 (0.78, 0.84) 0.623	.00021 (-.00024, .00067)	-.0032 (-.029, .022)			-.00052 (-.0018, .00074)	1.00 (0.90, 1.12) 0.982	
65+	-.00076 (-.00093, -.00059)	.0081 (.0019, .014)	.00026 (-.00001, .00054)	0.78 (0.75, 0.81) 0.237	-.000016 (-.00036, .00033)	.0041 (-.018, .027)			.00063 (-.00089, .0021)	1.15 (1.03, 1.30) 0.416	
Race/Ethnicity:											
White	-.00055 (-.00067, -.00043)	.0041 (-.0014, .0095)	.000083 (-.00017, .00034)	0.81 (0.78, 0.83) Ref.	.000030 (-.00044, .00049)	-.0014 (-.024, .021)			.000082 (-.00090, .0011)		1.00 (0.91, 1.11) Ref.
African American	-.00063 (-.0011, -.00018)	.0060 (-.011, .024)	-.000015 (-.00090, .00088)	0.84 (0.79, 0.89) 0.240	-.00019 (-.00071, .00032)	.029 (.0089, .051)			-.00026 (-.0014, .00093)		1.47 (1.11, 1.96) 0.013
Hispanic	-.00026 (-.00073, .00020)	-.015 (-.031, .00076)	-.00055 (-.0013, .00017)	0.67 (0.61, 0.74) 0.001	.0013 (.00039, .0022)	.030 (-.033, .094)			-.0064 (-.0095, -.0033)		1.22 (0.92, 1.61) 0.215
Other	-.0010 (-.0016, -.00039)	.018 (-.0088, .032)	.00051 (-.00079, .0018)	0.76 (0.68, 0.84) 0.253	.00036 (-.00062, .0013)	-.0015 (-.055, .052)			-.00058 (-.0044, .0032)		1.17 (0.81, 1.68) 0.437
Unknown	--	--	--	0.81 (0.75, 0.87) 0.970	--	--			--		0.98 (0.74, 1.31) 0.902
Charlson comorbidity index:											
≤2	-.00034 (-.00047, -.00021)	.0040 (-.0018, .0099)	.00014 (-.00021, .00049)	0.74 (0.66, 0.82) Ref.	.00014 (-.0018, .0021)	-.028 (-.12, .067)			-.0019 (-.0070, .0031)		0.86 (0.72, 1.02) Ref.
3-9	-.00063 (-.00075, -.00051)	.0038 (-.00082, .0084)	.000056 (-.00015, .00026)	0.79 (0.78, 0.81) 0.199	.00021 (-.000090, .00051)	.0011 (-.015, .018)			-.00018 (-.0010, .00067)		1.13 (1.03, 1.23) 0.007
≥10	-.0014 (-.0022, -.00076)	.063 (.033, .093)	-.00024 (-.0019, .0014)	1.01 (0.89, 1.14) <0.001	-.00066 (-.0016, .00026)	.019 (-.022, .062)			.0028 (-.00028, .0059)		1.90 (1.00, 3.62) 0.022
Type of admission:											
Elective	-.00073 (-.00098, -.00049)	.0038 (-.0076, .015)	.000063 (-.00044, .00056)	0.68 (0.65, 0.73) Ref.	.0018 (.00054, .0032)	-.084 (-.15, -.016)			-.0022 (-.0064, .0018)		0.89 (0.79, 1.00) Ref.
Non-Elective	-.00056 (-.00065, -.00047)	.0057 (.0013, .010)	.000022 (-.00022, .00027)	0.82 (0.80, 0.84) <0.001	-.000074 (-.00033, .00017)	.016 (.0052, .026)			.00043 (0.000, .00085)		1.38 (1.24, 1.53) <0.001
Primary health insurance:											
Medicare	-.00084 (-.0010, -.00067)	.010 (.0037, .017)	.00028 (-.00011, .00067)	0.77 (0.75, 0.80) Ref.	-.000049 (-.00036, .00026)	.00079 (-.018, .020)			.00065 (-.00061, .0019)	1.08 (0.95, 1.22) Ref.	
Medicaid	-.00031 (-.00049, -.00014)	.0033 (-.0059, .013)	-.00038 (-.00082, .000059)	0.88 (0.83, 0.93) <0.001	-.00029 (-.0010, .00043)	.027 (-.014, .068)			.00019 (-.0025, .0029)	1.29 (1.03, 1.62) 0.180	
Private insurance	-.00039 (-.00049, -.00029)	-.0025 (-.0082, .0032)	-.000040 (-.00035, .00027)	0.79 (0.76, 0.82) 0.488	.00044 (-.00012, .0010)	-.0072 (-.034, .019)			-.0011 (-.0021, -.00025)	1.00 (0.88, 1.13) 0.403	
Self-pay/No pay/Other	-.00069 (-.0011, -.00033)	.019 (.0032, .036)	-.00012 (-.00089, .00066)	0.86 (0.79, 0.95) 0.024	.00039 (-.00052, .0013)	-.0033 (-.062, .056)			.000077 (-.0033, .0035)	1.21 (0.86, 1.70) 0.546	

**Table 4:** Interrupted Time Series and Poisson Regression for Radiation Therapy and Stereotactic Radiosurgery Utilization – According to hospital characteristics* ^a^ Of all hospitalizations corresponding to patients who were diagnosed with brain metastases; ^b^ Of all hospitalizations corresponding to patients who were diagnosed with brain metastases and underwent radiation therapies. IRR: Incidence Rate Ratio; ITSA: Interrupted Time Series Analysis; PR: Poisson Regression. * Interrupted time-series and Poisson regression analyses are presented for the total population and within strata of patient and hospital characteristics.

	Radiation Therapy ^a^				Stereotactic Radiosurgery ^b^				
	ITSA β (95% CI)			PR	ITSA β (95% CI)				PR
	Time point effect	Post-ACA effect	Post-ACA by time effect	IRR (95% CI) P _interact_	Time point effect	Post-ACA effect	Post-ACA by time effect		IRR (95% CI) P _interact_
Hospital region:									
Northeast	-.00018 (-.00038, .000020)	-.012 (-.025, -.00013)	-.00018 (-.00079, .00044)	0.81 (0.78, 0.85) Ref.	-.00013 (-.0011, .00084)	-.028 (-.071, .014)		.0014 (-.00024, .0029)	0.78 (0.66, 0.92) Ref.
Midwest	-.00069 (-.00092, -.00046)	.016 (.0084, .023)	-.000018 (-.00032, .00028)	0.85 (0.81, 0.89) 0.177	.00099 (.00041, .0016)	-.025 (-.051, .00095)		-.00064 (-.0015, .00026)	1.21 (1.02, 1.44) <0.001
South	-.00056 (-.00073, -.00039)	.0042 (-.0034, .012)	-.000050 (-.00044, .00034)	0.79 (0.77, 0.83) 0.446	.00014 (-.00036, .00064)	.015 (-.0084, .039)		-.00049 (-.0017, .00066)	1.23 (1.08, 1.41) <0.001
West	-.00099 (-.0013, -.00074)	.018 (.0099, .027)	.00048 (.000082, .00088)	0.74 (0.69, 0.79) 0.017	-.00075 (-.0018, .00031)	.049 (-.0048, .10)		-.00072 (-.0036, .0021)	1.05 (0.86, 1.27) 0.023
Hospital location/teaching status:									
Rural	.00039 (.00015, .00065)	-.029 (-.042, -.015)	-.00049 (-.0011, .00017)	0.76 (0.68, 0.84) Ref.	.0012 (.00044, .0019)	-.070 (-.12, -.025)		.000030 (-.0016, .0016)	0.54 (0.29, 0.98) Ref.
Urban –Non-Teaching	-.00074 (-.00088, -.00059)	.018 (.012, .025)	-.00050 (-.00082, -.00019)	0.77 (0.74, 0.81) 0.737	.00064 (.00014, .0011)	-.0035 (-.031, .024)		-.0010 (-.0022, .00022)	1.37 (1.14, 1.65) 0.003
Urban –Teaching	-.00072 (-.00085, -.00060)	.0030 (-.0024, .0085)	.00043 (.00013, .00073)	0.79 (0.77, 0.82) 0.352	-.00033 (-.00081, .00014)	.010 (-.011, .032)		.00025 (-.00099, .0015)	0.97 (0.89, 1.07) 0.055
Hospital bed size:									
Small	-.00061 (-.00084, -.00038)	.0036 (-.0072, .014)	.00049 (.000031, .00096)	0.79 (0.73, 0.86) Ref.	-.00069 (-.0030, .0017)	.044 (-.018, .11)		-.0020 (-.0059, .0018)	0.81 (0.62, 1.06) Ref.
Medium	-.00057 (-.00082, -.00033)	.0050 (-.0052, .015)	-.00011 (-.00062, .00038)	0.75 (0.71, 0.79) 0.273	.00072 (.000049, .0014)	.0031 (-.050, .056)		-.0026 (-.0055, .00034)	1.09 (0.90, 1.31) 0.081
Large	-.00058 (-.00068, -.00048)	.0048 (-.00013, .0098)	.000086 (-.00013, .00030)	0.81 (0.79, 0.84) 0.503	.00011 (-.00031, .00054)	-.0052 (-.023, .013)		.00062 (-.00018, .0014)	1.09 (0.99, 1.19) 0.043

We observed ITS disparities after stratifying by each patient and hospital characteristic, separately. Although declining trend in radiation therapy was consistently observed in all sub-groups, post-ACA status was related to higher utilization only among patients who were female, 18-39 or ≥ 65 years of age, those with CCI ≥10, non-elective admissions, Medicare, self-pay, no pay or other insurance, and those admitted to Midwestern, Western and urban (non-teaching) hospitals, with a negative relationship between post-ACA status, and utilization among Northeastern and rural hospitals. Stratified ITS analyses for radiation therapy showed no statistically significant time by post-ACA status interactions, except for hospital region, location, and teaching status whereby trends in utilization differed pre-post ACA. ITS analyses of SRS showed that SRS prevalence increased over time among Hispanics, elective admissions, and rural hospitals. Furthermore, we observed that post-ACA status was associated with higher SRS utilization among African-Americans, non-elective admissions, and lower SRS utilization among elective admission and rural hospitals. Significant time by post-ACA status in the context of SRS utilization was observed in the context of Hispanics and private insurance only. Similar disparities by patient and hospital characteristics were observed in the context of stratified Poisson regression models. Whereas stratum-specific associations between post-ACA status and utilization of radiation therapy were consistently negative, post-ACA status was positively related to SRS utilization in the context of those ≥ 65 years, African Americans, with CCI > 2 as well as non-elective admissions, Medicaid recipients, Midwestern, Southern, and urban (non-teaching) hospitals. By contrast, post-ACA status was negatively related to SRS utilization among rural hospitals.

## Discussion

In this series of cross-sectional studies, we used multiple time points defined based on year and quarter of hospital admission from NIS (2005-2013) and ITS analyses to examine whether ACA may have influenced utilization of radiation therapies and SRS among hospitalized U.S. adult patients diagnosed with brain metastases. Key findings were as follows: 1) There was an overall decline in radiation therapy utilization with an upward shift in utilization post-ACA; 2) Post-ACA, radiation therapy utilization shifted downwards for Northeastern and rural hospitals and upwards for certain categories of patients (females, 18-39 or ≥ 65 years of age, CCI≥10, non-elective admissions, Medicare, self-pay, no pay or other insurance) and hospitals (Midwestern, Western regions, urban (non-teaching)); 3) There was no clear overall trend in SRS utilization, although there was an increasing trend in SRS utilization among Hispanics, elective admission and rural hospitals; 4) Post-ACA, African-Americans and non-elective admissions experienced upward shift whereas elective admissions and rural hospitals experienced a downward shift in SRS utilization. Although the general decline in utilization of radiation therapy could be attributed to changes in healthcare practices over time, post-ACA upward shifts in radiation therapy and SRS utilization may be attributed to expanded access to healthcare services through health insurance, especially among traditionally underserved populations, which include racial/ethnic minorities and patients with pre-existing conditions.

Previously conducted ITS analyses focused on the potential role of ACA on utilization of healthcare services have examined specific ACA provisions, including Medicaid expansion [28,29], elimination of cost-sharing expenses for preventive services [17,19], and overall ACA implementation [17,19]. Given the complexity of this issue, few studies specifically focused on cancer diagnosis, prevention and treatment pre-post ACA and these studies yielded inconsistent findings, with evidence for improved healthcare access among underserved populations [17,19]. For instance, Carlos et al. used patient-level analytic files on 1,763,959 commercially insured women aged 40 to 74 years from the 2004-2014 Clinformatics Data Mart to evaluate changes in mammography cost-sharing and utilization pre-post ACA, while comparing these outcomes by age group, and found limited role of ACA in cost-sharing and utilization due to simultaneous revision of USPSTF guidelines [26]. By contrast, Haakenstad performed ITS analyses using 2009-2012 data on commercially-insured and Medicare beneficiaries, 50-75 years, from the Maine Health Data Organization All-Payer Claims Database to estimate ACA impact on trends in rural-urban disparities in colonoscopy rates and costs, and found decline in urban-rural disparities attributed to ACA [18]. Using data on 44,343 insured individuals, 50-64 years, who participated in 2007-2015 Medical Expenditure Panel Survey, Mbah et al. performed ITS to estimate the role of ACA cost-sharing provision in ethnic disparities in colorectal cancer screening and found increased utilization that did not differ between Hispanics with non-Hispanic patients [19]. An ITS analysis was conducted by Steenland et al. using Massachusetts All-Payer Claims Database (2009-2012) to evaluate changes in cost and utilization trends for breast, cervical, and colorectal cancer screenings post-ACA [23]. Whereas ACA was associated with decline in weekly copayment for preventive breast and cervical cancer screenings, copayment for colon cancer screening declined but the decline rate slowed after ACA and there was weak evidence for ACA impact on increased rates of cancer screening [23]. Lu et al. performed ITS analyses using JPS Center for Cancer Care institutional registry data on 4,808 urban, underserved, adult patients diagnosed with a first primary invasive solid tumor between 2008 and 2015, to examine ACA in relation to stage at diagnosis in Texas, a Medicaid non-expansion state [24]. Their results suggested that ACA decreased the prevalence of uninsured cancer patients but had little effect on cancer stage at diagnosis [24]. Moss et al. examined ACA Medicaid expansion (2008-2010 vs. 2011-2014 and 2014 vs. 2011-2013) with insurance rates among 181,866 women diagnosed with cervical, uterine, or ovarian cancer using 2008-2014 SEER data [30]. The study found a significant increase in Medicaid enrollment after 2011, and a significant decrease in uninsured rates for all cancer types between 2011 and 2014 [30]. After January 2014, uninsured rates decreased by 50% for uterine and ovarian cancer and by 25% for cervical cancer [30]. These changes were noted only among U.S. States that expanded Medicaid coverage [30]. Similarly, our study findings suggest that the ACA implementation may have expanded access to healthcare services, in general, and preventive services, in particular, through insurance coverage of underserved populations, thereby increasing early-stage detection of cancer and potentially reducing the need for radiation therapy among hospitalized patients with brain metastases and increasing utilization of SRS, a relatively expensive technology, among hospitalized patients needing radiation therapy.

To our knowledge, this study is the first to examine the potential role of ACA implementation on utilization of radiation therapy and SRS using a large, all-payer, database at the national level. Study findings should, nevertheless, be interpreted with caution and in light of several limitations. First, we relied on an administrative database consisting of discharge records with limited scope and granularity. Particularly, hospital location was identified as U.S. region rather than U.S. State and county-level data were not available, precluding our ability to stratify discharge records according to implementation of the ACA Medicaid expansion or to quasi-experimentally evaluate variations in insurance coverage pre-post ACA using the difference-in-difference methodology. Furthermore, unlike the SEER-Medicare and NCD databases, the HCUP NIS database does not collect detailed information on cancer diagnosis, staging, and treatment. Second, complete subject analysis was performed with potential for selection bias because of missing data, especially with respect to race and ethnicity. Third, many study variables, including brain cancer diagnosis and treatment, were defined using ICD-9 codes, potentially leading to misclassification bias. Fourth, observed time trends may have alternative explanations besides ACA implementation, including secular trends as well as the economic recovery after 2008. In addition, residual confounding may have led to biased differences in outcome among distinct subgroups defined by patient and hospital characteristics. Fifth, the role of chance cannot be eliminated given the limited number of patients who underwent radiation therapy and SRS. Sixth, the cross-sectional design of each wave of NIS does not allow establishment of temporality or causal relationships between variables of interest. Finally, study results can only be generalized to hospitalized U.S. adults within the period of interest, whose characteristics may differ from those who sought outpatient care for SRS and/or non-SRS radiation therapies at later time points.

## Conclusions

Hospitalized U.S. adults with brain metastases utilized less radiation therapy and slightly more SRS over time between 2005 and 2013. However, utilization levels and trends were not consistent among distinct sub-groups defined by patient and hospital characteristics, with some traditionally underserved populations more likely to receive healthcare services post-ACA implementation. These results are consistent with some of the previously published studies and with the idea that the ACA may be helpful at reducing the need for radiation therapy as well as closing the gap in access to technologically advanced treatments for brain metastases such as SRS. Delays in cancer diagnosis and treatment may be responsible for health disparities whereby racial and ethnic minorities in the United States are more likely to present at later cancer stages to the healthcare system and to experience higher cancer-related morbidity and mortality rates as compared to whites. Future research is needed to explore the question of whether expanded health insurance, in general, and ACA, in particular, can promote earlier diagnosis and treatment of brain metastases, thus reducing morbidity and mortality among minorities and other underserved populations.
